# *JWA* gene regulates PANC-1 pancreatic cancer cell behaviors through MEK-ERK1/2 of the MAPK signaling pathway

**DOI:** 10.3892/ol.2014.2329

**Published:** 2014-07-09

**Authors:** YUAN-YUAN WU, TIE-LIANG MA, ZHI-JUN GE, JIE LIN, WEI-LIANG DING, JIA-KE FENG, SU-JUN ZHOU, GUO-CHANG CHEN, YONG-FEI TAN, GUO-XING CUI

**Affiliations:** 1 Department of Gastroenterology, The Affiliated Yixing Hospital of Jiangsu University, Yixing, Jiangsu 214200, P.R. China; 2 Central Laboratory, The Affiliated Yixing Hospital of Jiangsu University, Yixing, Jiangsu 214200, P.R. China; 3 Department of Critical Care Medicine, The Affiliated Yixing Hospital of Jiangsu University, Yixing, Jiangsu 214200, P.R. China; 4 Department of Cardiac and Thoracic Surgery, The Affiliated Yixing Hospital of Jiangsu University, Yixing, Jiangsu 214200, P.R. China; 5 Department of General Surgery, The Affiliated Yixing Hospital of Jiangsu University, Yixing, Jiangsu 214200, P.R. China

**Keywords:** JWA, PANC-1 cells, MAPK pathways, siRNA

## Abstract

The present study aimed to investigate the role of *JWA* gene in the proliferation, apoptosis, invasion and migration of PANC-1 pancreatic cancer cells and the effect on the MAPK signaling pathway. Human PANC-1 pancreatic cancer cells were cultured *in vitro*, and small interfering RNA (siRNA) was designed for the *JWA* gene. The siRNA was transfected into PANC-1 cells. Subsequently, the cell proliferation was measured by MTT assay; cell apoptosis was detected by analyzing BAX and Bcl-2 protein expression; cell migration and invasion were measured using Transwell^®^ chambers; and the protein expression of JWA and ERK1/2, JNK and p38 and their phosphorylated forms were measured by western blotting. By utilizing the MTT assay, the results showed that when JWA protein expression was inhibited, the proliferation of PANC-1 cells was enhanced. In addition, the expression of apoptosis-associated protein (AAP) BAX was substantially decreased, while the expression of the apoptosis inhibitor gene, *Bcl-2*, was significantly enhanced. Using Transwell chambers, it was found that the number of penetrating PANC-1 cells was significantly increased after transfection with JWA siRNA, suggesting that the migration and invasion of the cells was substantially increased. By studying the association between JWA and the MAPK pathway in PANC-1 cells, it was found that the expression of p-ERK1/2 of the MAPK pathway was significantly downregulated following JWA siRNA transfection. However, the expression levels of ERK1/2, JNK, p38, p-JNK and p-p38 showed no significant differences. In conclusion, it was shown that JWA affects the proliferation, apoptosis, invasion and migration of PANC-1 pancreatic cancer cells which could be attributed to effects on the expression of ERK1/2 in the MAPK pathway.

## Introduction

Pancreatic cancer is known to be difficult to diagnose in its early stages and to treat medically. Pancreatic malignant tumors are common, latent, highly lethal and extremely difficult to be surgically treated. A previous report has suggested that ~90% of patients succumb to the disease within one year after diagnosis while the five-year survival rate is <5% (1). The incidence of pancreatic cancer has been on the increase in recent years, thus demonstrating the importance of studying the pathogenesis.

Alterations in gene and protein expression, and the activation of signaling pathways, are associated with the occurrence and progression of pancreatic cancers (2). JWA, a newly identified tubulin-associated protein, encodes a cytoskeleton-associated protein (AF 070523, 1998) that shares similar biological functions with tubulin-associated proteins. JWA may regulate the tubulin and actin system, affect cell migration, and may be associated with the biological functions of a number of tumor promoters and inhibitors, and involve the corresponding signaling pathways (3). JWA appears to have a significant role in both the directed and non-directed tumor cell migration (2).

The MAPK signaling cascade is a highly conserved pathway that transfers extracellular signals to cellular proliferation signals. The MAPK pathway triggers a genetic signaling cascade to the nucleus, resulting in regulation of cell proliferation, differentiation, apoptosis, gene expression and cellular response to the external environment (4). The MAPK signal transduction pathways of cell proliferation, apoptosis, invasion and migration converge, an event that is significant to the occurrence and progression of hematopoietic malignancies, epithelial tumors and choriocarcinoma (5,6). There have been no previous reports as to whether JWA gene affects the proliferation, invasion and migration of pancreatic tumors through the MAPK pathway. The present study therefore provides. to the best of our knowledge, evidence for the first time for the treatment and prognosis of pancreatic cancer.

In order to determine the function of JWA in PANC-1 human pancreatic cancer cells, the expression level of the *JWA* gene was downregulated using JWA-specific small interfering RNA (siRNA). Subsequently the proliferation, apoptosis, invasion and migration of PANC-1 cells were analyzed. Additionally, the present study analyzed the association between these cell functions with the MAPK signaling pathway, in order to identify molecular mechanisms in the pathogenesis of pancreatic cancer.

## Materials and methods

### Cell culture

Human PANC-1 pancreatic cancer cells were purchased from ATCC and cultured in Dulbecco’s modified Eagle’s medium (DMEM) (HyClone, Logan, UT, USA) supplemented with 10% fetal bovine serum (FBS; Hangzhou Sijiqing Biological Engineering Materials Co. Ltd, Hangzhou, China), 100 U/ml penicillin and 100 mg/l streptomycin (Beyotime Institute of Biotechnology, Shanghai, China). The cells were grown at 37°C with 5% CO_2_ in a humidified incubator.

### JWA siRNA transfection

Human JWA-specific siRNA was purchased from Santa Cruz (sc-60874; Santa Cruz Biotechnology Inc., Santa Cruz, CA, USA). Transfections of siRNA were carried out using Lipofectamine^®^ 2000 (Invitrogen Life Technologies, CA, USA). The final concentration of siRNA used was 150 nM for a transfection period of 6 h. Cells were collected for subsequent analyses following 48 h incubation. Nonsense siRNA was used as a negative control (NC) and untransfected PANC-1 cells were used as a blank control.

### Measurement of cell proliferation by MTT assay

PANC-1 cell proliferation was measured by MTT assay in 96-well micro-culture plates. The cells were collected 5 h after transfection, and seeded at a density of 2×10^4^ cells/well in 96-well plates in DMEM containing 10% FBS. Five duplicate wells were set up for each group and the experiment was repeated three times. The PANC-1 untransfected and nonsense siRNA-transfected cells were used as controls. After 48 h incubation, 20 μl of 5 mg/ml MTT solution in phosphate-buffered saline, was added to each well for 4 h. The absorbance of each well was analyzed using an Infinite^®^ F50 Microplate Reader (Tecan Group Ltd., Männedorf, Switzerland) at a wavelength of 570 nm. Proliferation curves were plotted according to the optical density and the cell growth before and after transfection was compared.

### Measurement of cell invasion and migration by the Transwell^®^ assay

A cell invasion assay was performed using Transwell chambers. A volume of 100 μl Matrigel^®^ (BD Biosciences, Franklin Lakes, NJ, USA) was added to a 24-well Transwell chamber. An untreated Transwell chamber was used for the cell migration assay and a Matrigel-coated chamber was used for the cell invasion assay. A total of 100 μl cell suspension (diluted in DMEM) with a density of 2×10^5^ cells/ml, was added to the upper chamber while 600 μl DMEM with 10% FBS was added to the lower chamber. The chamber was incubated at 37°C for 24 h and then the non-migratory cells were subsequently removed from the upper surface of the filter using a cotton swab. The invasive cells that penetrated through the pores and migrated to the underside of the membrane were stained with 1% crystal violet solution for 15 min and then fixed using 4% paraformaldehyde. Nine random fields were counted for penetrating cells using a light microscope at ×200 magnification (Olympus BX41; Olympus Corporation, Tokyo, Japan).

### Western blotting

Total protein was extracted from PANC-1 cells using RIPA buffer (Beyotime Institute of Biotechnology) 72 h after transfection and 40 μg protein was separated by SDS-PAGE. Following electro-transfer of the proteins to a Hybond enhanced chemiluminescence (ECL) nitrocellulose membrane, the membrane was blocked using skim milk powder at room temperature (15–25°C) for 1.5 h. The membrane was then incubated at 4°C overnight with rabbit polyclonal BAX, Bcl-2 (Abcam, Cambridge, UK), phospho-p38, phospho-ERK1/2, phospho-JNK, phospho-MEK, p38, ERK1/2, JNK, and MEK (Cell Signaling Technology, Inc., Danvers, MA, USA) antibodies and mouse anti-human GAPDH monoclonal antibody (Beyotime Institute of Biotechnology), respectively. The membranes were then washed prior to incubation with secondary IgG antibody (Merck KGaA, Whitehouse Station, NJ, USA) labelled with alkaline phosphatase and visualized by ECL. The membranes were scanned and the relative level of protein expression was analyzed.

### Statistical analysis

Data were processed using SPSS 14.0. Data are presented as the means ± standard deviation, using Student’s t-tests or one-way analysis of variance. P<0.05 was considered to indicate a statistically significant difference.

## Results

### JWA-specific siRNA transfection downregulates JWA gene expression in PANC-1 cells

**Figure 1:**
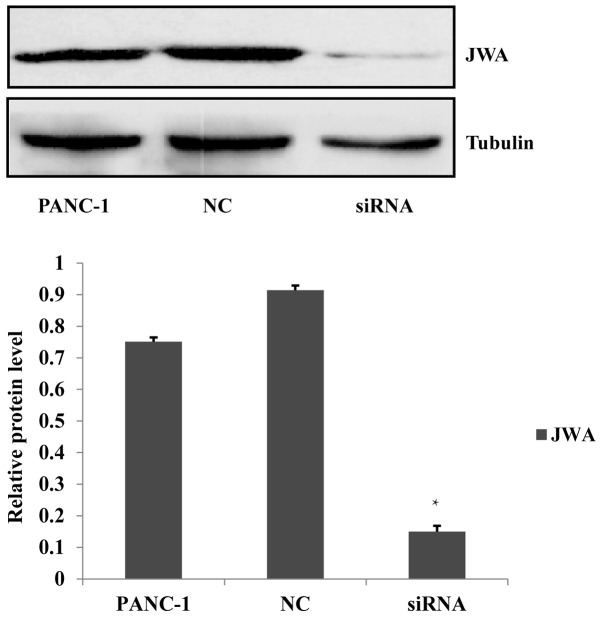
Effects of JWA siRNA. To investigate whether JWA was involved in PANC-1 cells, a specific JWA siRNA cell culture model was developed. The specific siRNA and the NC siRNA were transiently transfected into PANC-1 cells, respectively. Western blot analysis showed that the specific siRNA significantly suppressed the expression of JWA (^*^P<0.05) (siRNA group) as compared with the PANC-1 blank group and the NC. Error bars represent the standard deviation. siRNA, small interfering RNA; NC, negative control; PANC-1, human pancreatic cancer cells.

For the purpose of studying the association between the JWA and MAPK pathways in pancreatic cancer cells, an siRNA for JWA was prepared and transfected into PANC-1 cells. Nonsense siRNA transfected into PANC-1 cells was used as a NC and untransfected PANC-1 cells were used as a blank control. The protein expression, analyzed by western blotting, of JWA in PANC-1 cells transfected with JWA siRNA was significantly lower as compared with the negative and blank controls. This indicated that the JWA siRNA was effective in silencing the *JWA* gene and protein expression (Fig. 1). As a result, subsequent experiments investigating the effects of JWA knockdown should be performed using the JWA siRNA in PANC-1 cells.

### Cell proliferation following JWA siRNA-mediated knockdown

**Figure 2:**
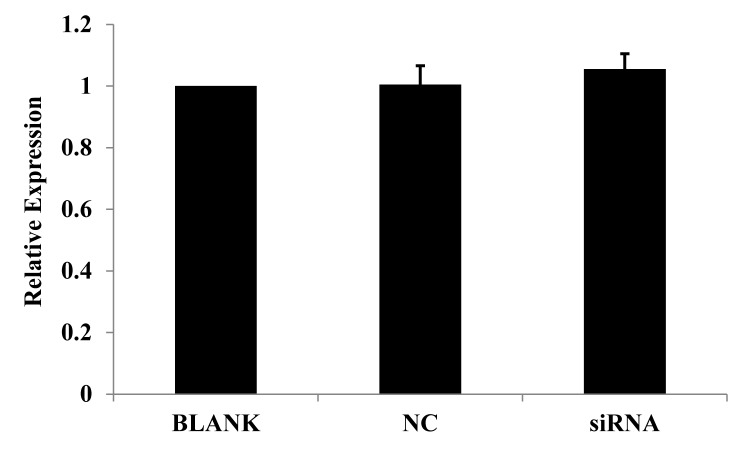
Effects of JWA siRNA on PANC-1 cell proliferation. PANC-1 cells were transfected with JWA siRNA and then seeded in 96-well plates and incubated with MTT following 24 h of transfection. The absorbance of each well was then measured. The proliferation of PANC-1 cells transfected by JWA siRNA was slightly higher than the other two groups, although the difference was not statistically significant (P>0.05). Data were normalized against the blank control set at 1. Error bars are the standard deviation. siRNA, small interfering RNA; NC, negative control; PANC-1, human pancreatic cancer cells.

A previous study has shown that all-trans retinoic acid (ATRA) is crucial in inhibiting the cell proliferation of HeLa cells (7). An MTT assay was therefore used to measure the proliferation of PANC-1 cells. Proliferation was observed to be enhanced following JWA siRNA transfection for 24 h, as compared with the NC and blank controls. The change in proliferation, however, was not significant (Fig. 2).

### The effect of JWA siRNA on the apoptosis of PANC-1 cells

**Figure 3:**
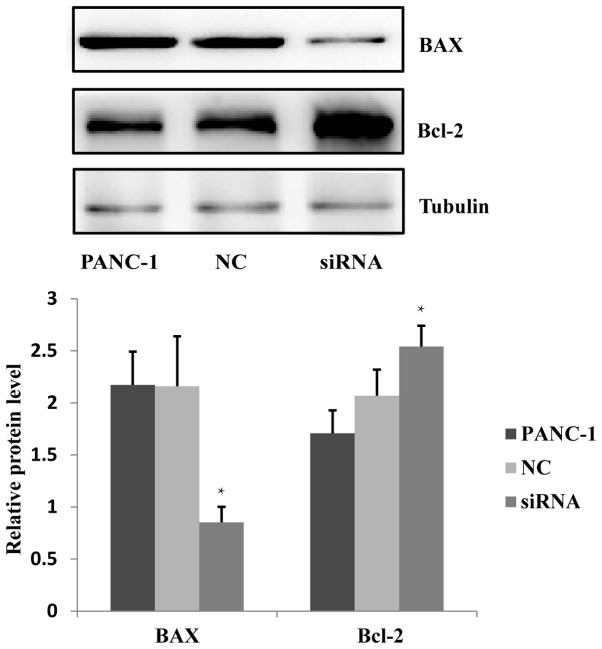
Effects of JWA siRNA on apoptosis. To determine the apoptotic effects of siRNA-mediated downregulation of JWA expression in PANC-1 cells, western blotting was used to examine the BAX and Bcl-2 protein expression in the siRNA, negative control and blank control groups. The western blotting showed that the expression level of BAX protein was significantly downregulated, whereas the expression level of Bcl-2 protein was increased, as compared with the blank (PANC-1) and NC groups. Error bars are the standard deviation, ^*^P<0.05. siRNA, small interfering RNA; NC, negative control. PNC-1, human pancreatic cancer cells.

Previous studies have indicated that JWA functions in the process of As_2_O_3_ and C/EBPα-induced apoptosis (8,9), and JWA overexpression has been shown to enhance the apoptosis of esophageal cancer cells (10). It has been additionally reported that the BAX protein expression in neoplasm of the digestive system, including liver and colorectal cancers, is downregulated and conversely, Bcl-2 protein is upregulated (11). The effects on apoptosis following JWA-specific siRNA transfection in PANC-1 cells was investigated. Western blotting was used to examine the BAX and Bcl-2 protein expression in cells treated with JWA siRNA, NC and blank control. The results indicated that the expression level of BAX protein was significantly downregulated and the expression level of Bcl-2 protein was increased (Fig. 3).

### The effects of JWA siRNA on the migration and invasion of PANC-1 cells by Transwell assay

**Figure 4:**
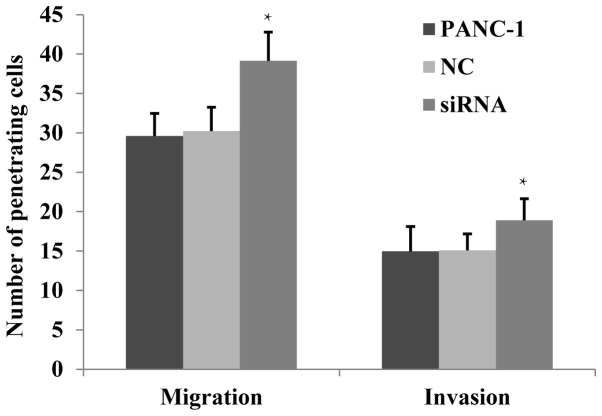
Effects of JWA siRNA on cell invasion and migration. Transwell chambers were used to detect cell invasion using Matrigel and cell migration without Matrigel. The cells migrating to the lower chambers were observed using a light microscope (magnification, ×200). The penetrating cell number of the JWA siRNA group was significantly increased in the non-basement membrane chamber and Matrigel-coated chamber, as compared with the NC and blank (PANC-1) group. The data suggest that when JWA expression was downregulated by siRNA, the migration and invasion of PANC-1 cells was significantly increased (^*^P<0.01). Error bars are the standard deviation. siRNA, small interfering RNA; NC, negative control; PANC-1, human pancreatic cancer cells.

It has been previously reported (10) that JWA downregulation enhances the migration of numerous tumor cells, whereas JWA overexpression inhibits cell migration. This suggests that JWA functions as a tumor suppressor gene (3). The migration and invasion ability of PANC-1 cells was analyzed using a Transwell assay. It was identified that the number of penetrating cells of the JWA siRNA-transfected group was found to be significantly increased (P<0.05) in both the non-basement membrane chamber and the Matrigel-coated chamber (Fig. 4). This suggested that following JWA expression downregulation, the migration and invasion of PANC-1 cells was significantly enhanced.

### The MEK-ERK1/2 pathway is activated following JWA knockdown

**Figure 5:**
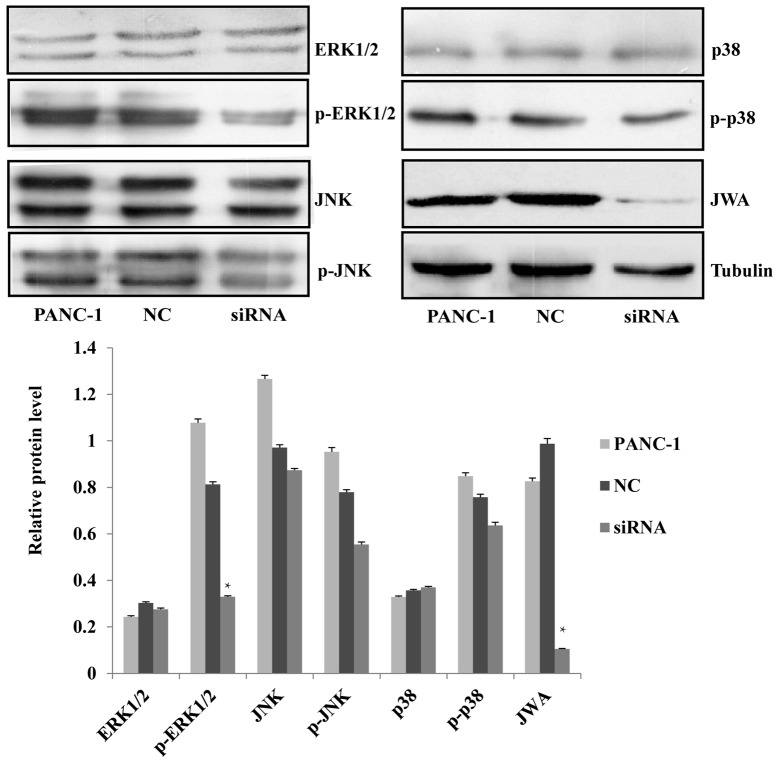
Western blot analysis of the key proteins of the three pathways of MAPK signaling. Western blotting showed that the expression of p-Erk1/2 was significantly downregulated, whereas the level of Erk1/2 showed no change. The expression of JNK and p38 and p-JNK and p-p38 were not significantly different. Error bars are the standard error. ^*^P<0.05. PANC-1, human pancreatic cancer cells; NC, negative control; siRNA, small interfering RNA; p, phosphorylated.

**Figure 6:**
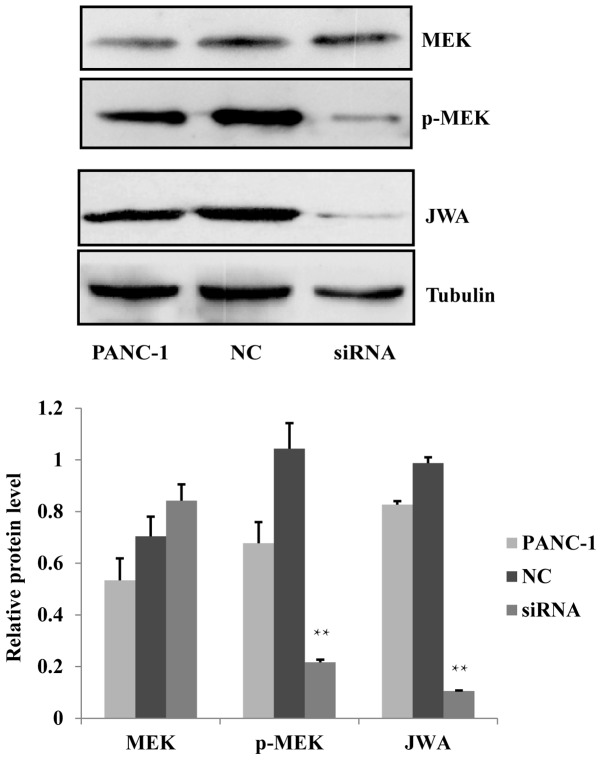
Western blotting of MEK protein expression. Western blotting revealed that the level of p-MEK was significantly decreased following JWA siRNA transfection, whereas MEK protein expression showed no significant difference. Error bars are the standard deviation. ^**^P<0.01 vs. PANC-1. PANC-1, human pancreatc cancer cells; NC, negative control; siRNA, smal interfering RNA; p, phosphorylated.

*Mao et al* (7) reported that inhibition of the proliferation and induction of apoptosis of HeLa cells by ATRA was due to the induction of ERK phosphorylation, while the downregulation of JWA inhibits ATRA-induced ERK phosphorylation (7). JWA is an essential factor of the Raf/MEK/MAPK signaling pathway, involved in the regulation of cell proliferation, apoptosis, migration, and invasion (3). The phosphorylated and non-phosphorylated forms of predominant proteins of the three MAPK pathways, were analyzed by western blotting. It was found that knockdown of JWA by siRNA resulted in the significant downregulation of p-ERK1/2 while the level of its non-phosphorylated form was not affected. The expression of JNK and p38, and their phosphorylated forms, was not significantly different (Fig. 5). It was observed that the protein expression level of the upstream factor of ERK1/2, p-MEK, was decreased after siRNA-mediated knockdown of JWA (Fig. 6). This suggested that the MEK-ERK1/2 pathway was activated and that the regulation of cell proliferation, apoptosis, migration and invasion may involve the MEK-ERK1/2 signaling cascade of the MAPK pathway.

## Discussion

The invasion and migration of tumor cells is a process subject to dynamic change, and is closely associated with the dynamic circulation of the cytoskeleton. A new cytoskeletal protein, JWA, was previously identified from the tissues of human primary tracheal and bronchial epithelial cells by Xu *et al* (12), who showed it regulates various biological functions including cell proliferation, differentiation and migration. In the present study, siRNA was used to knock down the expression of JWA in PANC-1 human pancreatic cancer cells and the association between JWA and the MAPK signal pathway was investigated.

Studies have identified that JWA is an important signaling molecule in the regulation of migration and differentiation of tumor cells, functioning as a tumor suppressor (10). In addition, JWA is associated with the occurrence and metastasis of malignant tumors (13). Studies carried out using liver cells with different metastatic potential have indicated that the higher the metastasis potential, the lower the expression of JWA mRNA and protein (14). Furthermore, previous studies have shown the downregulation of the expression of JWA protein in esophageal squamous cell carcinoma (ESCC) tissues, suggesting that JWA overexpression may inhibit the invasion and migration of tumor cells, including esophageal cancer cells (10,15). In the present study, the proliferation of PANC-1 cells was slightly enhanced, the protein expression of BAX was significantly decreased, and the expression of Bcl-2 was enhanced, following downregulation of JWA. Cell migration and invasion was significantly enhanced, which may be associated with cell proliferation and the involvement of JWA with cytoskeletal actin. The downregulation of JWA expression affected cell functions including migration, apoptosis and invasion.

MAPK signaling cascades are organized hierarchically into three-tiered modules, which are MAPK, MAPK-kinase (MAPKK) and MAPKK-kinase (MAPKKK). In eukaryotic cells, there are downstream-associated pathways, which include ERK1/2, JNK and p38 regulating cell proliferation, differentiation, development, apoptosis and inflammation (16). The MAPK signaling pathways are closely associated with the proliferation, apoptosis, invasion and migration of tumor cells, and are of great importance with tumor development and proliferation (17). It has been shown that PMA and As_2_O_3_ induce migration and invasion of tumor cells by activating the MAPK signaling pathways, and is associated with the process of reconstruction of cytoskeletal actin filaments (18). A previous study has shown that the MAPK pathways have a significant role in the development and differentiation of ovarian cancer caused by KRAS and BRAF mutations (19). The study by Yao *et al* (20) on breast cancer, suggested that the phosphorylation level of ERK1/2 in breast cancer cells was substantially higher as compared with normal breast cells, suggesting that the overexpression of ERK1/2 protein is of great importance in the occurrence and progression of breast cancer (20). The data of the present study have shown that the protein expression level of p-ERK1/2 and its upstream MAPKK factor p-MEK, was significantly decreased following JWA knockdown by siRNA. The expression level of ERK1/2, MEK and the other two pathways showed only slight changes.

These data indicate that PANC-1 cells may function through the MEK-ERK1/2 pathway of MAPK signaling cascades to regulate proliferation, apoptosis, invasion and migration.

In conclusion, the *JWA* gene has a significant function in the proliferation, apoptosis, invasion and migration of PANC-1 human pancreatic cancer cells. The increase of *JWA* gene expression may inhibit the invasion and migration of pancreatic cancer cells and this function may be achieved through the MEK-ERK1/2 pathway of the MAPK signaling cascades. These findings provide new scientific evidence to facilitate the clinical treatment of pancreatic cancer.
